# Defective Sphingolipids Metabolism and Tumor Associated Macrophages as the Possible Links Between Gaucher Disease and Blood Cancer Development

**DOI:** 10.3390/ijms20040843

**Published:** 2019-02-15

**Authors:** Marzena Wątek, Ewelina Piktel, Tomasz Wollny, Bonita Durnaś, Krzysztof Fiedoruk, Ewa Lech-Marańda, Robert Bucki

**Affiliations:** 1 Department of Hematology, Institute of Hematology and Transfusion Medicine, Indiry Gandhi 14, 02-776 Warsaw, Poland; ewamaranda@wp.pl; 2 Department of Microbiology and Immunology, The Faculty of Medicine and Health Sciences of the Jan Kochanowski University in Kielce, Aleja IX Wieków Kielc 19, 25-317 Kielce, Poland; Bonita.Durnas@onkol.kielce.pl; 3 Department of Microbiological and Nanobiomedical Engineering, Medical University of Bialystok, Mickiewicza 2C, 15-222 Bialystok, Poland; ewelina.piktel@wp.pl (E.P.); buckirobert@gmail.com (R.B.); 4 Holy Cross Cancer Center, Prezydenta Stefana Artwińskiego 3, 25-734 Kielce, Poland; tomwollny@gmail.com; 5 Department of Microbiology, Medical University of Bialystok, Mickiewicza 2C, 15-222 Bialystok, Poland; krzysztof.fiedoruk@umb.edu.pl

**Keywords:** Gaucher disease, sphingolipids, cancer, tumor-associated macrophage, multiple myeloma

## Abstract

There is a rising number of evidence indicating the increased risk of cancer development in association with congenital metabolic errors. Although these diseases represent disorders of individual genes, they lead to the disruption of metabolic pathways resulting in metabolite accumulation or their deficiency. Gaucher disease (GD) is an autosomal recessive sphingolipidosis. It is a rare lysosomal storage disease. A strong correlation between GD and different types of cancers, such as multiple myeloma, leukemia, and hepatocellular carcinoma, has been reported. Common features for all types of GD include spleen and liver enlargement, cytopenia, and a variety of bone defects. Overall, the molecular bases leading to the association of GD and cancers are not clearly understood. Here, we describe the role of ceramides in GD, discuss the potential implications of immune cells activation and show how the disturbances in their metabolism might promote blood cancer development.

## 1. Introduction

Carcinogenesis is often described as a process that involves eight basic features, namely (i) self-sufficient proliferation, (ii) insensitivity to pro-apoptotic and anti-proliferative signals, (iii) achievement of unlimited replication potential, (iv) proper vascularization of tumor formation, (v) metastatic capacity, (vi) invasion of neighboring tissues, (vii) reprogramming of energy metabolism and (viii) evading immune destruction. In addition to cancer cells, the repertoire of seemingly normal recruited cells that residue without tumor mass is important, since they create a “tumor microenvironment” that contributes to the tumor’s acquisition of characteristic features. Reactive microenvironment, tumor stroma with an abundance of inflammatory mediators from associated cells (leukocytes, macrophages) has a huge impact on the regulation of angiogenesis, promotion of cancer cell proliferation and continuous rotation of the matrix and repression of adaptive immunity. All these factors ultimately affect the progression of the disease [1,2]. Cancer growth can also be placed in the context of different development phases: initiation, promotion, and progression. Initialization is characterized by genetic changes leading to irreversible cellular alterations such as point mutations, deletions, or rearrangements of chromosomes. The development of a tumor is promoted by the survival and expansion of “initiated” cells. Progression refers to a significant increase in a tumor size and its ability to propagate via metastases [1]. In GD, congenital enzyme acid β-glucosidase (glucosylcerebrosidase; GlcCerase) deficiency results in the accumulation of glucocerebroside (glucosylceramide) in the organelles, late endosomes and lysosomes of macrophages (known as Gaucher cells, GCs), that are observed in many organs, mainly in bone marrow, spleen, liver, and lymph nodes [3]. So far, two mechanisms that can increase tumorigenesis in the course of Gaucher’s disease were postulated. According to the first one, disturbed cellular and cytokinic local microenvironmentin GD is the major culprit, since GCs are chronically, alternatively-activated macrophages secreting several proinflammatory and anti-inflammatory cytokines, chemokines, and hydrolases [4]. Whereas, the alternative hypothesis assumes that tumorigenesis results from inappropriate balance between pro- and anti-proliferative sphingolipids occurring in the (future) malignant cell itself. It is known that ceramide, the product of GlcCerase activity, is a strong tumor suppressor, strengthens the signals initiating apoptosis, autophagy, and cell cycle arrest [5], and its decrease contributes to the prolonged survival of cancer cells. In addition, as observed in a mouse model, the accumulation of glucosylceramide leads to the activation of an alternative metabolic pathway of sphingolipids, in which under the influence of the second active GlcCerase at neutral pH (*GBA2* gene), sphingosine is produced, followed by pro-proliferative sphingosine-1-phosphate (S1P) [6].

## 2. Gaucher Disease

**Figure 1:**
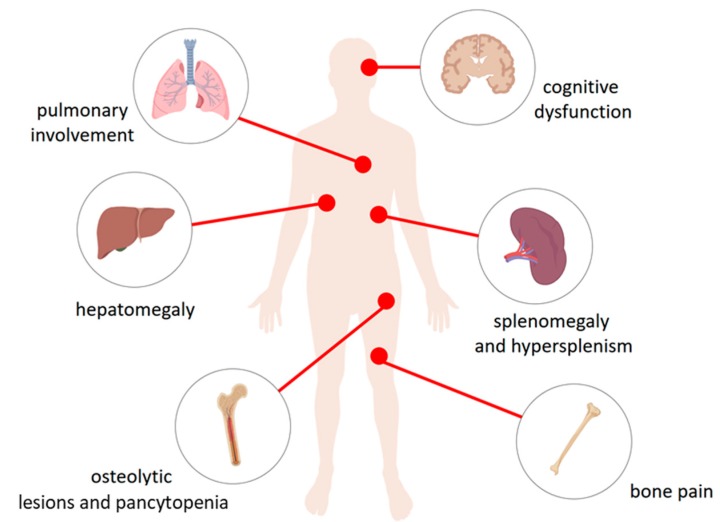
The physiological consequences of accumulation of glucocerebroside in the lysosomes of the reticuloendothelial system cells. Macrophages loaded with glucocerebroside (Gaucher cells) infiltrate various tissues and organs to varying degrees depending on the type of disease and causing their dysfunction. They affect the liver causing hepatomegaly, spleen—splenomegaly and hypersplenism, bone and bone marrow—osteolytic lesions and pancytopenia in type 1, 2, and 3 disease, bone pain as well as the central nervous system in the types Neuropathic 2 and 3 causing cognitive dysfunction. Gaucher cells were also recorded in lungs.

GD is a congenital lysosomal storage disorder (Table 1) [7,8]. The deficiency of the enzyme results from the biallelic *GBA1* mutation, and more than 300 mutations in the gene encoding the lysosomal hydrolase were identified [9]. In connection with deficiency of the enzyme, sphingolipid metabolism is disturbed and accumulation of non-used substrate (glucocerebroside) take place [7,8] (Figure 1). A characteristic histological manifestation of GD is the accumulation of the Gaucher cells (GCs). These are macrophages containing glucosylceramide with characteristic appearance of “wrinkled paper”. This is due to the cell filling through the lysosomal storage structures of glucosylceramide within the cytoplasm [10]. Based on the macrophage model THP-1 GCs, it was concluded that the primary site of glucosylceramide (GlcCer) accumulation were lysosomes. As more GlcCer accumulated, they spread evenly throughout the cell and they are distributed among all subcellular fractions [11,12]. There were also observed secondary elevations in the concentrations of sphingolipids: ceramide, phosphatidylglycerol, phosphatidylinositol, phosphatidylcholine, sphingomyelin, di-, and trihexosylceramide [11,12].

Three types of GD are described. Type 1, found in the majority of patients (90% in Europe and the USA, less in other regions), is characterized by enlargement of the internal organs [6] that include splenomegaly, hepatomegaly, cytopenia’s, complex bone involvement with necrosis, osteoporosis, fractures, and lytic changes [7,8,10]. Extremely rare type 2 (acute neuronopathic) and type 3 (sub-acute neuronopathic), except symptoms present in type 1 disease, are associated with severe neurological disorders in type 2 or variables in type 3 [6,10]. The complex multi-systemic phenotype arises due to glucocerebroside accumulation in macrophages of the liver, spleen, bone marrow, and sometimes lung in GD type 1; neuronopathic fulminant in type 2 (GD2), and chronic neuronopathic progress in type 3 (GD3). In 1991, enzyme replacement therapy (ERT) was introduced to treat the disease. This therapy has become the standard of care that changed the natural history of patients with GD [7,8]. GD can very rarely be caused by a deficiency of saposin C (SapC) [13] that is one of the four homologous proteins derived from the sequential cutting of prosaposin—a saposin precursor protein. SapC is an essential activator of glucocerebrosidase, with described deficiency in GD [14]. In Kang et al. case description, the patient was clinically presented as the typical form of type 1 disease with more frequent manifestations including hepatosplenomegaly, thrombocytopenia, and anemia. The presence of Gaucher cells was found in the bone marrow. In the plasma of patients, the activity of chitotriosidase [13] (an enzyme produced by activated macrophages (GCs)), a glucocerebroside storage marker [15] and glucosylsfingosine were also highly elevated, which was confirmed by the diagnosis of the Gaucher disease. However, it was surprising that the leukocyte β-glucosidase activity was normal. The reason for this observation was the detection of an innovative maternal c.1133C > G mutation (p. Pro378Arg) in exon 10 of the prosaposin precursor protein (PSAP) gene. This gene encodes the SapC domain of the PSAP protein. The authors concluded that the biallelic mutations of the PSAP gene are the cause of the patient’s Gaucher disease. Discovery of the spectrum of mutations of the Gaucher disease with a deficiency of saposin C expanded the knowledge about the GD pathophysiology [13]. It is generally accepted that the severity of GD depends on the level of the remaining enzyme activity. Although genotype-phenotype correlations are weak, it is known that some mutations predispose to the type of disease. The homozygosity of L444P causes a neuronopathic disease, whereas the presence of even one mutant allele for N370S prevents neurological involvement. Remarkably, phenotypes may differ even in siblings or monozygotic twins [16,17]. It was shown that the deacylated form of glucosylceramide, glucosylsphingosine, is significantly increased in the plasma of patients with GD type 1 symptoms (*n* = 64, median = 23.7 nM, range 15.6–5.25 nM, normal (*n* = 28): median 1.3 nM, range 0.8–2.7 nM). It was further found that plasma glucosylsphingosine levels correlate with established GCs plasma markers, chitotriosidase (ρ = 0.66) and CCL18 (ρ = 0.40) [18].

## 3. Cancers Frequency/Incidence in Gaucher Disease

Within a few decades, many cases of GD1 (the dominant type of disease) coexisting with hematological malignancies were described [19]. Those cases have increased our knowledge of their interplay. Overall, the most common described examples of GD1 association with cancers are monoclonal gammopathy of undetermined significance (MGUS) [20] and hematological malignancies (multiple myeloma) [10]. The most frequently described hematological cancer is multiple myeloma, and less often chronic lymphocytic leukemia, acute lymphoblastic leukemia, and myeloid neoplasms (chronic myelogenous leukemia, acute myelogenous leukemia). The association of GD with solid tumors of brain, bone, colon, kidney, liver, prostate, testis, and melanoma were also described [10]. However, it should be noted, that for some researches the thesis about more frequent prevalence of cancers in patients with GD than in the general cancer population is controversial. For example, Ilan et al. in a retrospective study involving a group of 505 patients diagnosed with GD1 in Israel showed that only 20 patients (4%) developed cancer. The most common were lymphomas (three cases) and myelodysplastic tumours (three cases). Myeloma was diagnosed in many subjects as well. Likewise, a higher prevalence of these diseases was not observed in comparison to the frequency in the population of healthy Israeli Jews. Also the results obtained in the study of the international GD registry (ICGG) did not confirm the increased incidence of cancer in patients with GD in addition to multiple myeloma. The estimated relative risk of myeloma was 5.9 compared to 0.79 in the healthy population. In a population of 1525 veterans with GD, there was an increased risk of non-Hodgkin’s lymphoma, melanoma and pancreatic cancer [21]. Increased incidence of hematopoietic malignancies B cell lymphoma/myeloma and monoclonal gammopathy in authentic murine model of Gaucher disease with a modest accumulation of β-glucosylceramide but greatly increased de-acylated form—β-glucosylsphingosine, suggesting that a bioactive lysolipid β-glucosylsphingosine plays a role in downstream signaling [22]. Furthermore, it was shown that inhibition of de novo synthesis of glycosphingolipids by eliglustat prevented development lymphoma and myeloma in mice with Gaucher disease and strikingly, long-term pharmacological inhibition of glycosphingolipids synthesis prevented development of early marker of multiple myeloma—monoclonal gammopathy [23].

In 1982, Lee et al. on the basis of post mortem examination of patients with GD1 showed the presence of cancer in 19 of 20 examined subjects and the most common cancer was multiple myeloma [24]. Likewise, Shiran et al. [19] on a small group of 48 patients with GD showed a significant difference in occurrence of neoplasms, 20.8%, in comparison to 6.8% (35 out of 511) in the group without GD diagnosis (*p* = 0.0027, relative risk 3.6). Furthermore, an increased risk of hematopoietic tumors by 10.4% in the patients with GD compared to 0.78% in the control group (*p* = 0.00037) was reported [19]. Finally, the study of cancer patients included in the International Gaucher Registry (2742 patients with all types of GD but over 90% of patients were diagnosed with type 1 disease) shown that a single diagnosis of cancer was reported in 126 patients, but some patients were diagnosed with more than one neoplasm [7].

## 4. Pathogenesis of Cancer Associated with GD

### 4.1. Sphingolipids

Sphingolipids, similarly to other membrane lipids, are considered as building blocks of the cell biological membranes as well as molecules with well-known effects on cell growth, differentiation, migration, and apoptosis [25]. The sphingomyelin-signaling pathway was pronounced as the third transmembrane signaling route in 1987 by Kolesnick et al. [26,27,28]. Ceramide, bioactive transmitter of the sphingomyelin signal transduction pathway shows pro-apoptotic and tumor suppressor activities. Its degradation by the ceramidase results in formation of sphingosine, phosphocholine and sphingosine-1-phosphate (S1P) [29]. S1P, another bioactive transmitter regulates many physiological and pathophysiological processes including cancer formation, inflammation, atherosclerosis, diabetes, and osteoporosis [30,31]. A broad spectrum of biological effects of these compounds arises from the diversity of the receptors associated with the sphingomyelin signaling pathway. Molecular mechanisms of ceramide action involve the activation of the enzymes (kinases) participating in proteins phosphorylation/dephosphorylation. Ceramide-activated protein kinases are usually membrane-specific proteins such as CAPK (Ceramide-activated protein kinase) and CAPP (Ceramide-activated protein phosphatase). Also other protein kinases, namely ceramide-activated protein MAPK kinase (Mitogen-Activated Protein), protein kinase Cζ and JNK kinase (c-jun- N-terminal protein kinase), are under sphingomyelin signal transduction pathway control (13, 14). The pro-apoptotic function of ceramide involves triggering enzymes such as caspase 3 and 8 that participate in breakdown of the cell cytoskeleton [32,33]. On the other hand, ceramide tumor suppressor activity is associated with induction of cancer cells apoptosis and slowing tumor growth by transmitting apoptotic signals through T lymphocytes. In addition, inhibition of ceramide production, either pharmacological or molecular, impairs production of interleukin-2 (IL-2) and programmed cell death after activation of T cell-induced receptor (TCR) [34,35]. It is worth to underline that ceramide 1-phosphate (C1P), the phosphorylated form of ceramide, has opposite properties, i.e., mitogenic and prosurvival. For instance, Mitra et al. observed that in vitro low concentration of C1P increases survival of A549 lung carcinoma cells and NIH 3T3 fibroblasts, while high concentration reduces their survival and causes apoptosis [24]. The latter phenomenon can be explained by degradation of C1P to pro-apoptotic ceramide [24]. Therefore, the balance between pro-apoptotic ceramide (pro-apoptotic sphingolipids) and anti-apoptotic C1P (pro-proliferative sphingolipids) was proposed as a mechanism of cell fate determination [24].

Presently, there is very limited data about the abnormal ceramide concentrations in patients diagnosed with Gaucher disease. Nevertheless, a study by Groener et al. suggests that alterations in ceramide metabolism might considerably affect the GD-related cancers development. Although plasma ceramide concentrations were not significantly different between the healthy patients and subjects with GD, in individual patients plasma GlcCer/Cer ratio allowed for better discrimination between Gaucher disease patients and normal individuals than the GlcCer level alone. Importantly, plasma GlcCer concentration and GlcCer/Cer ratio were positively correlated with severity of disease, plasma chitotriosidase and CCL18 [36].

Interestingly, carriers/scavengers of S1P and ceramide, such as human plasma gelsolin (pGSN), were recently proposed to function as the important factors involved in development of hematological malignancies [37]. Since pGSN has ability to interact with broad spectrum of cellular mediators including S1P or PAF, it contributes to regulation of inflammatory and cancer-promoting processes [38,39,40]. Our previous study aiming to evaluate the sphingolipid profile in blood and bone marrow plasma of AML patients demonstrated the correlation between low levels of pGSN and altered blood concentrations of ceramide and S1P. These observations suggest that the regulation of S1P and ceramide-mediated signaling pathways may be beneficial in development of novel anti-AML therapies [37].

### 4.2. Deficiency of Acid β-Glucosidase, Immune Pathology in Gaucher Disease

As aforementioned, acid β-glucosidase is responsible for the lysosomal hydrolysis of glucocerebroside (GlcCer, also known as glucosylceramide) to ceramide. Therefore, its deficiency in patients with GD leads to progressive accumulation of glucocerebroside and its deacylated product, glucosylsphingosine, in macrophages known as Gaucher cells (GCs). Remarkably, recent studies indicate a strong influence of glucosylsphingosine (or Lyso-glucosylceramide) on cell dysfunction in GD, and thus its usefulness as a novel biomarker of GD [6,41]. In addition, an accumulation of secondary bioactive sphingolipids associated with the ceramide metabolism was observed. For example, sphingosine-1-phosphate (S1P), that is involved in pro-proliferative, pro-mitogenic, anti-apoptotic, and pro-angiogenic signaling. On the other hand, a possible deficiency of ceramide, which is an important effector in the regulation of autophagy and apoptosis [42], exerts opposite effects. Finally, secondary to the metabolic block, there is accumulation of Cer, phosphatidylglycerol (PG), trihexosylceramide (THC), and diheksosylceramide (DHC) [43].

GlcCer has been shown to be presented to NK lymphocytes (NKT) and dendritic cells (DCs) via CD1 molecules. It can exert immunomodulatory effects through mechanisms that have been shown in studies indicating change in NKT cells plasticity, promotion of regulatory T cells (T reg), change in the composition of lipid rafts and intracellular signaling [21].

It should be mentioned that GCs are not just mobile warehouses of glucosylceramide excess, but they are chronically activated macrophages responsible for a variety of GD’s clinical symptoms. For example, GCs lead to an increase of plasma levels of many proinflammatory and anti-inflammatory cytokines, chemokines, and hydrolases [4]. Hence, plasma levels of massively secreted chitotriosidase and CCL18 (chemokine, ligand 18) are used to monitor efficacy of enzyme substitution in GD therapy (ERT) [44]. Moreover, in serum collected from GD1 mouse model, elevated levels of IL-13 and IL-4 have been observed, suggesting an increase in activity of GD1 macrophages [10]. Factors released by GCs, including interleukin-1β (IL-1β), interleukin-1 receptor antagonist, soluble IL-2 receptor (sIL-2R), tumor necro sis factor-α (TNF-α), macrophage colony-stimulating factor (MCSF) and IL-6 that indicate macrophage activation also are elevated in the serum of Gaucher patients [45]. Those cytokines’ response causes chronic B-cell stimulation. Indeed, extremely high prevalence of gammopathies in GD patients was observed [46]. Additionally other pro-inflammatory and anti-inflammatory cytokines and growth factors are out of hemostatic range in GD [10]. Potentially changes in cytokines may explain some of the GD pathology. IL-1β, IL-6, TNF-α, and IL-10 may cause osteopenia, IL-6 and IL-10 gammopathies and lead to multiple myeloma [47]. Altogether, these effects create an environment that promotes tumorigenesis [10].

### 4.3. Lipid Rafts (Detergent-Resistant Membranes, DRM)

The mosaic model of cell membrane construction proposed by Singer and Nicolson in 1972, after decades of functioning, in the context of innovative discoveries, including the functionality of the signaling cell membrane, had to be changed. The high density of proteins in the bilayer makes it a “crowded” molecularly space with important physiological consequences. Essential in the context of sphingolipidosis are membrane heterogeneities (membrane domains) called lipid rafts with the possibility of localized and transient lipid (flip-flop) movement [48]. Lipid rafts are sets of sphingolipids and cholesterol in the cell membrane as well as in organelle-vascular membranes, e.g., Golgi complex, endoplasmic reticulum, or mitochondria. They are very dynamic structures, constantly and quickly changing their composition and playing an important role in various biological processes. They are inhomogeneous membrane domains with a high content of non-particulate glycolipids in detergents [49]. It is believed, that it performs many cellular functions including proliferative and apoptotic information. The lipid composition of DRM in GD is altered as a direct consequence of the inability of the cell to degrade GlcCer. In the macrophage model of Gaucher disease, it was shown that in addition to increased cellular concentrations of GlcCer, Cer, DHC, and THC were also increased. GlcCer accumulation occurs in DRM by replacing phosphatidylcholine (PC), the most abundant lipid in DRM. There were no changes in cholesterol concentrations. The data suggest that the function of membrane rafts in macrophages of Gaucher disease is impaired due to changes in the composition of phospholipids and sphingolipids [43].

Lipid rafts are involved in combining actin with a cell membrane. Changes in the lipid composition of rafts lead to the rearrangement or relaxation of actin-membrane bonds with the resulting changes of the actin cytoskeleton and as a consequence of “stuttering of the endosomal system” [50].

Endocytosis plays important functions in the normal cell physiology, but also has a significant role in pathology. One of the basic, well-described functions of endocytosis is the uptake of nutrients, uptake of cholesterol by the LDL receptor or iron by transferrin and transferrin receptor. An important role of endocytosis is the regulation of the expression of membrane proteins, especially receptors and transporters. The decisive factor in regulating the surface expression levels of membrane proteins is the balance between recycling/smuggling to storage organelles or late endosomes and lysosomes (LE/Ly). Membrane sorting that occurs in endosomes is important for the regulation of cell physiology [51].

### 4.4. Chitotriosidase

Chitotriosidase (Chitinase 1, CHIT1) is the major, active chitinase in the human body, belonging to the family of 18 glycosyl hydrolases that is mainly produced by activated macrophages [52,53]. Members of this family also include various chitin-like proteins, such as Chi3l1/YKL-40, Ym1 and Ym2 Overall CHIT1 is the best characterized chitinase. It gains recognition as a key component of the innate host defense mechanism against both bacterial and fungal infections [53]. The activity of CHIT1 in the healthy population is very low and comes from circulating polymorphonuclear cells [54]. CHIT1 might be considered as a prognostic biological marker of GD and its concentration correlates with the disease burden. Additionally, chitotriosidase is useful in monitoring GD therapy (enzymatic replacement therapy, ERT). It reflects the “alternative” type macrophage activation, including Gaucher cell [55].

CHIT1 is also perceived as an innate immune mediator, able to digest the cell walls of chitin-containing eukaryotic pathogens, e.g., fungi. CHIT1 serum concentration strongly correlates with the progression or the severity of many diseases, such as tuberculosis, idiopathic pulmonary fibrosis, scleroderma-associated interstitial lung diseases (SSc-ILD), sarcoidosis and chronic obstructive lung diseases (COPD) [53]. Chitotriosidase is currently recognized as a gravity screening marker and/or therapeutic monitor for over 40 different diseases, both inherited and acquired. Most of those diseases are associated with the activation of macrophages and the production of the chitotriosidase. However, CHIT1 is a nonspecific marker of macrophage activation, limiting the utility of its assessment as a screening marker for specific clinical situations. Though, as mentioned before in established diagnoses of GD, it is an excellent indicator for monitoring response to treatment [52]. Increasing evidence demonstrated that chitinases and chi-lectins, especially CHI3L1 (YKL-40), could play a key role in human cancer development, associated with bad prognosis and increased tumor angiogenesis [52,56] including non-small cell lung cancer (NSCLC) [56], invasive cervical cancer (CxCa) [57], breast, ovarian or colon cancer [58]. Also, in patients diagnosed with Philadelphia negative/Ph (−) myeloproliferative cancer (MPN), it was shown that CHIT1 was significantly higher in the case of polycythemia vera (PV) (*p* < 0.001) and bone marrow transformation (MF) after PV (MFP after PV). There was no higher level of CHIT1 in essential thrombocythemia ET, transformation after MF after and primary myelofibrosis (PMF), compared to healthy controls [59]. It is worth noting that many analyses indicate a strong relationship between YKL-40 and tumor activity, its progression, and propensity to create metastases and a shorter survival time. The probable mechanism of this phenomenon is the operation of YKL-40 through the signal pathways that involves FAK and PI3K/AKT engagement [58].

### 4.5. Tumor Associated Macrophages (TAM)

A connection between microenvironment, signal path disorder and development or metastatic tumor was often suggested in the past. The microenvironment of solid tumors is characterized by a reactive stroma with an abundance of inflammatory mediator, leukocytes, fibroblasts, and vascular endothelial cells as well as dysregulated vessels formation and large spectrum of proteolytic enzymes activity [60,61].

Tissue macrophages display a variety of biological activities that depend on their location and signals received from the environment [62]. Based on differences in activating stimuli and expression of surface and/or cytoplasmic markers, macrophage populations were divided into classically (M1 phenotype) and alternatively (M2 phenotype) activated. The expression of receptors and macrophage-fragmented molecules is influenced by microenvironmental factors [63].

Macrophages that constitute a heterogeneous population constitute up to 50% of the tumor mass and play an important role in tumor pathophysiology, especially in response to changes in the local microenvironment. They exhibit different molecular phenotypes and release different cytokines, inhibiting (M1) or promoting (M2) tumor growth [35].

It is now widely accepted that TAMs have the M2 phenotype and possess tumor promoting functions, namely cell survival, proliferation, and dissemination. High levels of TAMs are often correlated with poor prognosis, and recent studies highlight the relationship between their abundance and the metastasis process [60]. Hence, TAMs are considered as “protumoral macrophages” and major players in the connection between inflammation and cancer, that connect a number of functions (e.g., promotion of tumor cell proliferation and angiogenesis, incessant matrix turnover, repression of adaptive immunity), which ultimately have an important impact on disease progression. Thus, together with other myeloid-related cells present at the tumor site (Tie2 macrophages and MDSCs—myeloid derived suppressor cells), TAMs represent an attractive target of novel biological therapies of tumors [60].

The implications of TAMs in Gaucher disease pathophysiology are still relatively unexplored, to date. Nevertheless, some studies suggest that Gaucher cells are phenotypically related to tumor-associated macrophages. Most importantly, research performed by Ivanova et al. demonstrated that cells observed in the extraosseous Gaucheromas (i.e., tumor-like structures formed in the results of Gaucher cells conglomeration) are characterized by cellular morphology similar to atypical GCs. Deeper analysis revealed that these atypical cells exhibited the properties of tumor-associated macrophages, since positive expression for CD163, CD68, and VEGF, a well-known TAMs markers, was recorded. Above observations encouraged the suggestion that Gaucher cells attract migration of other macrophages and promote differentiation toward TAM, supporting the pseudotumors formation and development [64]. Similar suggestions were made by other authors [10]. We believe that TAM properties of GD might contribute to the increased frequency of hematological and solid tumors in patients with Gaucher disease, nevertheless this issue needs to be more thoroughly investigated.

## 5. What Induces Cancerogenesis in GD?

In 1863, Virchow developed the hypothesis that cancer arises in places of chronic inflammation. This theory is based on the observations that some classes of molecules, along with tissue damage and the resulting inflammation, increase cell proliferation. Certainly, the promotion of cancer risk is due not only to cell proliferation but also due to prolonged cell proliferation in an environment containing inflammatory cells, cytokines, activated stroma, and agents promoting DNA damage [65,66].

The immune system has evolved mechanisms that protect the host against the development of cancer. The impairment of control mechanisms allows the development of cancer. In the studies in mice it was shown that macrophages, NK cells, T lymphocytes, as well as cytokine damage or neutralization including INF-gamma or Interleukin 12 (IL-12) increase the host’s susceptibility to cancer. In humans, the proof of this hypothesis is the aggravated increase in tumorigenesis in people with congenital and acquired (eg transplantation) immune deficiencies [67].

GCs display a highly heterogeneous expression pattern for CD163, which is specific for alternatively activated macrophages (M2 type) [4]. Macrophages are known to be versatile cells and they can react to stimuli in a variety of ways [4]. The patients with GD have increased concentrations of many blood cytokines and chemokines [68]. However, they do not produce typical proinflammatory molecules such as TNF-α, IL-12p40 and interferon-γ, or MCP-1 (monocyte chemoattractant protein 1, known as CCL2), but have a clear expression of anti-inflammatory IL-1Ra. Part of the GCs express also IL-6, that has recently been described as an important factor in the induction of MCP-1 and in the recruitment of monocytes [4]. All variants of the GD show the involvement of monocytes, macrophages, dendritic cells, T lymphocytes and B lymphocytes [68]. A lot of evidence confirms the involvement of cytokines in the initiation, promotion, invasion, and metastasis of cancer. Inflammatory cytokines, including TNF-α and IL-6 or chemokines (e.g., IL-8), induce the production of free radicals, trigger invasiveness, support angiogenesis, and the ability to create metastasis. They can also damage the DNA causing mutations that lead to tumor initiation [69]. GD and the accompanying changes (disorders of the sphingolipid signaling pathway and GCs/TAMs) may constitute a model of tumor pathogenesis. It is known, that the deficiency of pro-apoptotic ceramide affects TAMs [35], It was proved experimentally on the mouse model, that injection of liposome-loaded with C6-ceremide (LipC6) induce TAMs—M2 macrophage to differentiate into an M1 macrophage phenotype. LipC6 injection in mice with liver tumors reduces the number of TAMs and their ability to suppress the immune anti-tumor response. LipC6 also increases the anti-tumor effect of CD8^+^ T lymphocytes [70]. It increases anti-cancer protection.

**Figure 2:**
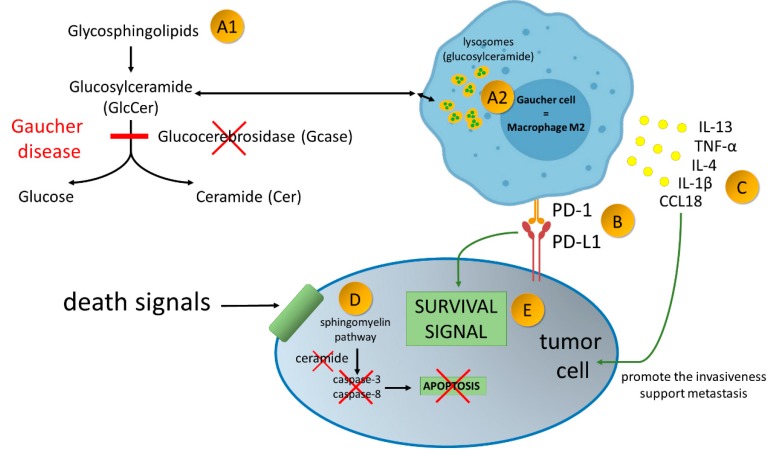
Gaucher disease—an inherited metabolic disorder caused by mutations in the gene *GBA1*, encoding acid-β-glucosidase (GlcCerase; Gcase)–A1. It leads to accumulation glucocerebroside in macrophages–A2. Gaucher Cells resemble alternatively activated macrophages (M2). Alternatively activated macrophages/Gaucher cells, play a pro-tumoral role in tumor microenvironment. PD-1 expression on macrophage cells affects cancer cell survival by binding to PD-L1 on the tumor cell–B. Production of broad spectrum of cytokines promote the invasiveness of cancer cells and support angiogenesis and their ability to create metastasis–C. Due to enzymatic block of ceramide formation, cell death signals transmitted to the cancer cell are not read–D. As the result of current disorders in the cancer cell, survival signals over ventricular death signals prevail–E.

Cancers in GD can also be caused by increasing tolerance of newly formed tumor cells, since in both mouse and human TAMs (presumably and GCs) express the programmed cell death protein 1 (PD-1), which is the resistance control point receptor. When up-regulated on activated T cells it induces immune tolerance. Tumor cells often overexpress the ligand for PD-1, the programmed cell death ligand 1 (PD-L1), which assures tolerance within the immune system [71]. The expression of TAM PD-1 increases over time in mouse cancer models as well as with the stage of the disease in primary human cancers. In addition, expression of TAM PD-1 correlates negatively with the phagocytic power of antineoplastic cells [71]. The potential mechanism of protumorigenic properties of Gaucher cells and implication of expression of PD1 on activated macrophages in malignancies development is presented in Figure 2.

PD1 is responsible for the regulatory role in this response. PD-1 receptor and the programmed cell death receptor, are the negative regulators of the immune response. They interact with PD-L1 and PD-L2 ligands and are responsible for maintaining peripheral immunoreactivity limits, proliferation and effectors function of T-lymphocytes. The expression of PD-1 and its ligands is described in many cancers, where it modulates tumor microenvironment. Hence, it has been proposed as a potential escape mechanism of cancer cells from immune surveillance [72]. Additionally, in vivo experiments indicate that blocking of PD-1-PD-L1 results in increases of macrophage phagocytosis, reduces tumor growth and prolongs mouse survival in mouse cancer models in a macrophage-dependent manner. This finding suggests that PD-1-PD-L1 targeting therapies may also work when directly affect macrophages [71]. Monoclonal antibodies that block the interaction between PD-1 and PD-L1 by binding to a ligand or receptor have demonstrated significant clinical efficacy in patients with various cancers including melanoma, colon cancer, non-small cell lung cancer, and Hodgkin’s lymphoma [71].

The change in the properties of lipid rafts mediated through the composition of phospholipids, adversely affects the location of rafts receptors and signaling in information routes. It has been proven that raft disruption disturbs IL-6/STAT3 and IFN-g/STAT1 signaling [21]. In addition, GlcCer has been shown to immunomodulate lymphocyte killer (NKT) and dendritic cells (DCs) through the CD1d cell differentiation antigen. Since, CD1d is also located in lipid rafts then changes in their composition may inhibit the activation of NKT cells despite the lack of weakening of CD1-ligand binding [21]. The human cluster of the CD1 differentiation system, the antigen presenting molecule to T lymphocytes consists of four types of antigen presenting molecules, each of which has a distinct functional niche: CD1a, CD1b, CD1c, and CD1d. CD1 molecules, using endosomes, present T lymphocytes with glyco- and phospholipid antigens [73]. It has been proven that inhibition of GlcCer disintegration in GD causes improper targeting of endosomes to lysosomes. The abnormal sorting of endocytes of macrophages in GD is due to the increased endolysosomal pH caused by the excess GlcCer. It is also known that pH levels in endosomes has an important role in lysosomal hydrolases activation [74].

We know that the most common type of cancer in patients diagnosed with GD is multiple myeloma [24]. Multiple myeloma from the phase of “pre-cancer” monoclonal gammopathy of uncertain importance (MGUS) through the asymptomatic phase progresses to a clinically active symptomatic disease. Myeloma is associated with an increase in immune dysfunction [41].

Interactions between myeloma cells in the bone marrow and bone marrow stromal cells (BMSCs) are important determinants of disease pathogenesis. Immunohistochemistry of samples collected from 22 patients with myeloma showed abundant infiltration of CD68^+^, CD16^+^, S100-negative macrophages accompanied by dispersed CD3^+^ T lymphocytes [75]. Additionally, TAMs, CD68^+^ with dendritic cell morphology constituted 2–12% of nucleated cells, and evenly distributed in the pulp where each dendrite was in a close contact with many plasma cells. In some cases, TAMs were strikingly concentrated around the blood vessels CD34^+^ [75]. Primary human CD14^+^ monocytes infected with oncolytic measles virus and administered intravenously to mice with KAS6/1 human myeloma xenografts prolonged survival of mice with disseminated human myeloma. Thus, TAMs are a universal framework component for plasmocytoma of patients with multiple myeloma and may be a promising new target for therapeutic use [75]. Understanding the antigenic responsiveness of a clonal immunoglobulin may allow understanding of the antigenic origin of myeloma, but may also lead to new strategies for the prevention and treatment of cancer by targeting the antigen-stimulating disease [41]. For example, Nair et al. evaluated whether or not monoclonal immunoglobulin is present in the context of Gaucher’s disease, and demonstrated that monoclonal antibodies (monoclonal gammopathy) were specific against glucosylsphingosine (or Lyso-glucosylceramide, LGL1). Hence, LGL serves as an antigenic target in the monoclonal gammopathy associated with Gaucher disease. Furthermore, in the mouse model of Gaucher disease, they observed that LGL sphingolipid mediates the activation of B cells and plasma cells. Therefore, antigenic reactivity of clonal immunoglobulin shows the antigenic origin of monoclonal gammopathy and myeloma. In gammopathy in patients with Gaucher disease, substrate reduction - LGL1, can be achieved by means of therapy to reduce substrate concentration [41]. Furthermore, glycosphingolipids have been shown to influence the activation of osteoclasts and the development of bone disease in myeloma. The dominant glycosphingolipid produced by myeloma cells from the patient and MM cell lines is GM3 (monosialodihexosylganglioside). The activation and development of osteoclasts by disrupting RANKL-induced tumor necrosis factor receptor associated factor 6 (TRAF6) and proto-oncogene tyrosine-protein kinase Src (c-SRC) in lipid rafts is prevented by inhibitors of glucosylceramide synthase (N-butyl-deoxynojirimycin (NB-N DNJ)). The addition of GM3 synergistically enhanced the ability of RANKL pro-osteoclastogenic agents and insulin-like growth factor 1 (IGF-1). The effect of osteoclast activation in mice increased GM3 administration and inhibited the administration of NB-DNJ. NB-DNJ in the mouse model of MM improved the symptoms of bone disease. The obtained data show that both glycosphingolipids of tumor origin and de novo synthesized influence osteoclastogenesis. The results suggest that NB-DNJ may be used clinically to reduce the pathological activation of osteoclasts and reduce bone destruction associated with MM [76].

In summary, there are many reasons for predisposition of patients with GD to cancer development: (i) bioactive lipids accumulation, (ii) GCs - alternatively activated macrophages, (iii) immune deregulation (accumulation of glucosylceramide)/PD1 expression/impaired NK cell activity, cytotoxicity of T lymphocytes, abnormal cytokine activity, impaired function of macrophages, and (iv) involvement of genetic modification (cancer phenotype, genetic mutation) [10].

## 6. Conclusions

Recognizing GD as a stage predisposing for the development of neoplastic diseases, strongly suggests request for monitoring of sphingolipids and pro-inflammatory cytokines concentration in affected subjects. Overall sphingolipids and their metabolites may serve as novel diagnostic marker to assess risk of cancer development. These attributes require further detailed scientific analysis.

## Figures and Tables

**Table 1:** Pathophysiology of Gaucher Disease.

Pathophysiology of Gaucher Disease		
Gene expression error	Biallelic mutation GBA1 gene (glucocerebrosidase 1 gene)	Biallelic mutation of the PSAP gene (prosaposine prekursor gene)
Consequence of gene mutation	Lysosomal deficiency of glucocerebrosiadase	Saposin C deficiency SAP = sphingolipid activator
Glucocerebrosidase activity	No activation of glucocerebrosidase	No activation of glucocerebrosidase
Accumulation of glucosylceramide	Within the organelles, late endosomes and lysosomes	Within the organelles, late endosomes and lysosomes

## Abbreviations

C1P	ceramid-1-phosphate
CAPK	ceramide-activated protein kinase
ERT	enzyme replacement therapy
GCs	Gaucher cells
GD	Gaucher disease
GlcCer	glucosyloceramide
MCP-1	monocyte chemoattractant protein 1, known also as CCL2
MGUS	monoclonal gammopathy of undetermined significance
MM	multiple myeolma
S1P	sphingosine-1-phosphate
